# Multiple and High-Risk Clones of Extended-Spectrum Cephalosporin-Resistant and *bla*_NDM-5_-Harbouring Uropathogenic *Escherichia coli* from Cats and Dogs in Thailand

**DOI:** 10.3390/antibiotics10111374

**Published:** 2021-11-10

**Authors:** Naiyaphat Nittayasut, Jitrapa Yindee, Pongthai Boonkham, Teerapong Yata, Nipattra Suanpairintr, Pattrarat Chanchaithong

**Affiliations:** 1 Department of Veterinary Microbiology, Faculty of Veterinary Science, Chulalongkorn University, Bangkok 10330, Thailand; 6175522431@student.chula.ac.th (N.N.); jitrapa.y@chula.ac.th (J.Y.); 2 Veterinary Diagnostic Laboratory, Faculty of Veterinary Science, Chulalongkorn University, Bangkok 10330, Thailand; pongthat.b@chula.ac.th; 3 Biochemistry Unit, Department of Physiology, Faculty of Veterinary Science, Chulalongkorn University, Bangkok 10330, Thailand; teerapong.y@chula.ac.th; 4 Department of Pharmacology, Faculty of Veterinary Science, Chulalongkorn University, Bangkok 10330, Thailand; nipattra.d@chula.ac.th; 5 Research Unit in Microbial Food Safety and Antimicrobial Resistance, Faculty of Veterinary Science, Chulalongkorn University, Bangkok 10330, Thailand

**Keywords:** *bla*_CTX-M_, carbapenemase, pet, clones, uropathogenic *Escherichia coli*

## Abstract

Resistance to extended-spectrum cephalosporins (ESC) and carbapenems in *Escherichia coli* (*E. coli*), increasingly identified in small animals, indicates a crisis of an antimicrobial resistance situation in veterinary medicine and public health. This study aimed to characterise the genetic features of ESC-resistant *E. coli* isolated from cats and dogs with urinary tract infections in Thailand. Of 72 ESC-resistant *E. coli* isolated from diagnostic samples (2016–2018), *bla*_CTX-M_ including group 1 (CTX-M-55, -15 and -173) and group 9 (CTX-M-14, -27, -65 and -90) variants were detected in 47 isolates (65.28%) using PCR and DNA sequencing. Additional antimicrobial resistance genes, including plasmid-mediated AmpC (CIT and DHA), *bla*_NDM-5_, *mcr-3*, *mph*(A) and *aac(6′)-Ib-cr*, were detected in these isolates. Using a broth microdilution assay, all the strains exhibited multidrug-resistant phenotypes. The phylogroups were F (36.11%), A (20.83%), B1 (19.44%), B2 (19.44%) and D (4.17%), with several virulence genes, plasmid replicons and an integrase gene. The DNA fingerprinting using a repetitive extragenic palindromic sequence-PCR presented clonal relationships within phylogroups. Multiple human-associated, high-risk ExPEC clones associated with multidrug resistance, including sequence type (ST) 38, ST131, ST224, ST167, ST354, ST410, ST617 and ST648, were identified, suggesting clonal dissemination. Dogs and cats are a potential reservoir of ESC-resistant *E. coli* and significant antimicrobial resistance genes.

## 1. Introduction

Urinary tract infection (UTI) is commonly caused by opportunistic bacteria and requires systemic antimicrobial treatment in small animal practice. *Escherichia coli* (*E. coli*) is a major pathogen causing UTIs in humans and animals, with a prevalence of 35–69% in dogs and cats [1]. According to the treatment guidelines developed by the International Society for Companion Animal Infectious Diseases (ISCAID), extended-spectrum cephalosporins (ESC), such as cefpodoxime, cefovecin and ceftiofur, are considered second-line antimicrobials for bacterial cystitis based on antimicrobial susceptibility results [2]. Due to the emerging spread of extended-spectrum β-lactamases (ESBLs), such as cefotaximase (CTX-M) and plasmid-mediated AmpC cephalosporinases (pAmpC) associated with multidrug resistance (MDR) [3,4,5], the available antimicrobial options have become ineffective for treatment. Extensively drug-resistant and pan drug-resistant Gram-negative bacteria, which are resistant to last-resort carbapenems and colistin, are a serious burden in human medicine [6] and have emerged in small animals and veterinary settings [7,8]. Mobilisable carbapenemase-encoding genes such as *bla*_NDM-5_ (New Dehli metallo-β-lactamase) and *bla*_OXA-181_ (oxacillinase) and *mcr* (mechanism of colistin resistance) genes in *E. coli* have been characterised and increasingly reported by animal sources worldwide [7,9,10]. Pets and people associated with pets having identical ESBL-producing *E. coli* strains support evidence of interspecies transmission of resistant bacteria between humans and animals [11].

Regarding zooanthroponotic transmission, high-risk *E. coli* clones in terms of sequence type (ST) by multilocus sequence typing (MLST) and virulent phylogroups are regarded as a public health issue. [5,12,13]. Uropathogenic *E. coli* (UPEC) is an extraintestinal pathogenic *E. coli* (ExPEC) pathotype mainly classified into virulent-phylogroup B2, containing numerous virulence factors for colonisation, invasion and survival outside intestine. High-risk human-associated ESC-resistant ExPEC ST38, ST131, ST410 containing *bla*_CTX-M_ group 1 and 9 have been identified in human clinical samples from Thailand [14]. Of these, pandemic ESBL-producing *E. coli* ST131 and carbapenemase-producing *E. coli* ST410 distributed in companion animals have been increasingly reported worldwide [9,15], but information about the genetic background and relatedness of *E. coli* to a present linkage from the companion animal sector as a part of the One Health concept is unavailable in Thailand. Based on our routine diagnostic antimicrobial susceptibility testing results, ESC and carbapenem resistance in *E. coli* associated with UTIs in dogs and cats have frequently been observed, and questions about the molecular epidemiological relationships among these isolates and with the clones distributing in animals and humans from different regions were raised. Therefore, the aims of this study were to characterise the genetic features and antimicrobial genotypes/phenotypes of canine and feline ESC-resistant UPEC and to present multiple human-associated ExPEC clones from dogs and cats in Thailand.

## 2. Results

### 2.1. Numbers and Sources of E. coli

Between 2016 and 2018, 344 *E. coli* isolates (9.99%) were isolated from 3442 clinical samples, including 2498 canine and 945 feline samples, submitted to the Veterinary Diagnostic Laboratory of Faculty of Veterinary Science, Chulalongkorn University. Samples from UTIs were the main source of *E. coli* isolates (43.9%, 151/344), followed by abscesses and subcutaneous tissue infections (16.86%, 58/344), surgical site infections (9.88%, 34/344), an abdominal cavity or fluid (9.88%, 34/344), the gastrointestinal tract (5.23%, 18/344), reproductive organs (4.94%, 17/344), otitis (2.91%, 10/344), nares (2.91%, 10/344) and unidentified sources (6.39%, 22/344). Seventy-two of the 151 *E. coli* isolates from UTIs that exhibited resistance to cefpodoxime, ceftiofur and/or cefovecin by the Vitek^®^ automated system were included for further characterisation. Of the 72 isolates, 31 (43.06%) expressed the ESBL phenotype by combination disk test (CDT).

### 2.2. ExPEC Virulence Genes

All of the 72 ESC-resistant *E. coli* isolates (100%) contained at least two virulence genes associated with extraintestinal infections. The *fimH* (type I fimbriae) was detected in all the isolates (100%, 72/72). The isolates carried ExPEC virulence genes in different proportions, including *crl* (curli (97.22%, 70/72)), *iucD* (aerobactin siderophore (55.56%, 40/72)), *papC* (P pili (22.22%, 16/72)), *cnf1/2* (cytotoxic necrotising factor (16.67%, 12/72)), *sfa/foc* (S fimbrial adhesin/F1C fimbriae (15.28%, 11/72)), *hlyA* (hemolysin A (15.28%, 11/72)), *sat* (secreted autotransporter toxin (15.28%, 11/72)), *iha* (iron-regulated gene homolog adhesin (13.89%, 10/72)), *iroN* (siderophore outer membrane receptor (11.11%, 8/72)) and *afa/dra* (Dr/Afa adhesins (4.17%, 3/72)). Only *vat* (vacuolating autotransporter toxin) was absent in all the isolates. The virulence gene profiles of the isolates are illustrated in Supplementary Table S1. Based on the presence of the ExPEC-associated virulence genes and isolation from UTI, UPEC could be deduced for the *E. coli* isolates [16].

### 2.3. Antimicrobial Resistance Phenotypes

All the ESC-resistant UPEC isolates displayed resistance to amino-penicillins (ampicillin), first-generation cephalosporins (cefalexin and cefazolin), ESCs (cefotaxime, cefpodoxime and cefovecin), anti-pseudomonal penicillins (piperacillin and ticarcillin) and sulfonamides (sulfamethoxazole). Resistance to additional β-lactams including cefoxitin (56.94%, 41/72), amoxicillin/clavulanic acid (51.39%, 37/72), ticarcillin/clavulanic acid (43.06%, 31/72), meropenem (5.56%, 4/72) and imipenem (5.56%, 4/72) was observed. All the UPEC isolates had MDR profiles, which expressed resistance to three or more antimicrobial classes (Supplementary Table S1). Resistance to fluoroquinolones, including nalidixic acid (93.06%, 67/72), enrofloxacin (90.28%, 65/72), ciprofloxacin (90.28%, 65/72) and marbofloxacin (88.89%, 64/72), was high. The resistance proportions of other antimicrobials were variable, including tetracycline (91.67%, 66/72), doxycycline (83.33%, 60/72), gentamicin (76.39%, 55/72), amikacin (9.72%, 7/72), trimethoprim (75.00%, 54/72), sulfamethoxazole/trimethoprim (76.39%, 55/72), chloramphenicol (61.11%, 44/72) and azithromycin (37.50%, 27/72). Six isolates (8.33%) showed resistance to colistin, four of which presented high-level resistance (MIC > 16 µg/mL). Five isolates (6.94%) and seven isolates (7.45%) showed resistance and intermediate resistance to nitrofurantoin, respectively. Intermediate resistance to enrofloxacin (4.16%, 3/72), ciprofloxacin (2.78%, 2/72), tetracycline (2.78%, 2/72), doxycycline (9.72%, 7/72), amikacin (20.83%, 15/72) and chloramphenicol (13.89%, 10/72) was observed among the UPEC population.

### 2.4. Antimicrobial Resistance Genes

Of the 72 isolates, 47 isolates contained *bla*_CTX-M_ (65.28%) consisting of *bla*_CTX-M-14_ (22.22%, 16/72), *bla*_CTX-M-55_ (16.67%, 12/72), *bla*_CTX-M-15_ (13.89%, 10/72), *bla*_CTX-M-27_ (9.52%, 4/72), *bla*_CTX-M-65_ (2.78%, 2/72), *bla*_CTX-M-173_ (2.78%, 2/72) and *bla*_CTX-M-90_ (1.39%, 1/72). The CIT and DHA types of pAmpC were detected in 29 isolates (40.28%) and 4 isolates (5.56%), respectively. Forty-three isolates (59.72%) carried *bla*_TEM_, and 20 isolates (27.78%) carried *bla*_OXA-1-like_. Plasmid-mediated quinolone resistance (PMQR) *aac(6′)-Ib-cr* gene and azithromycin resistance *mph*(A) gene were found in 21 isolates (29.17%) and 26 isolates (36.11%), respectively, whereas other PMQR genes, including *qnrA*, *qnrB*, *qnrC*, *qnrD*, *qnrS* and *qepA*, were not detected. One colistin-resistant isolate was *mcr-3*-positive, but no *mcr* gene was found in the other three isolates. The *bla*_NDM-5_ was identified in four carbapenem-resistant isolates.

### 2.5. Integrase Gene and Plasmid Replicons

Nine plasmid replicons were detected in this study. The F, FIB, FIA and I1-Iγ plasmids were the predominant types and detected in 55 (76.39%), 52 (72.22%), 37 (51.39%) and 20 (27.78%) isolates, respectively. Other replicons were sporadically found, including N (8.33%, 6/75), A/C (6.94%, 5/72), Y (4.17%, 3/72), FIC (2.78%, 2/72) and P (1.39%, 1/72). Forty-two UPEC isolates (58%) had the *intI*1 gene. The plasmid replicon types detected in the UPEC isolates and the presence of *intI*1 are illustrated in Supplementary Table S1.

### 2.6. Molecular Characteristics

**Figure 1:**
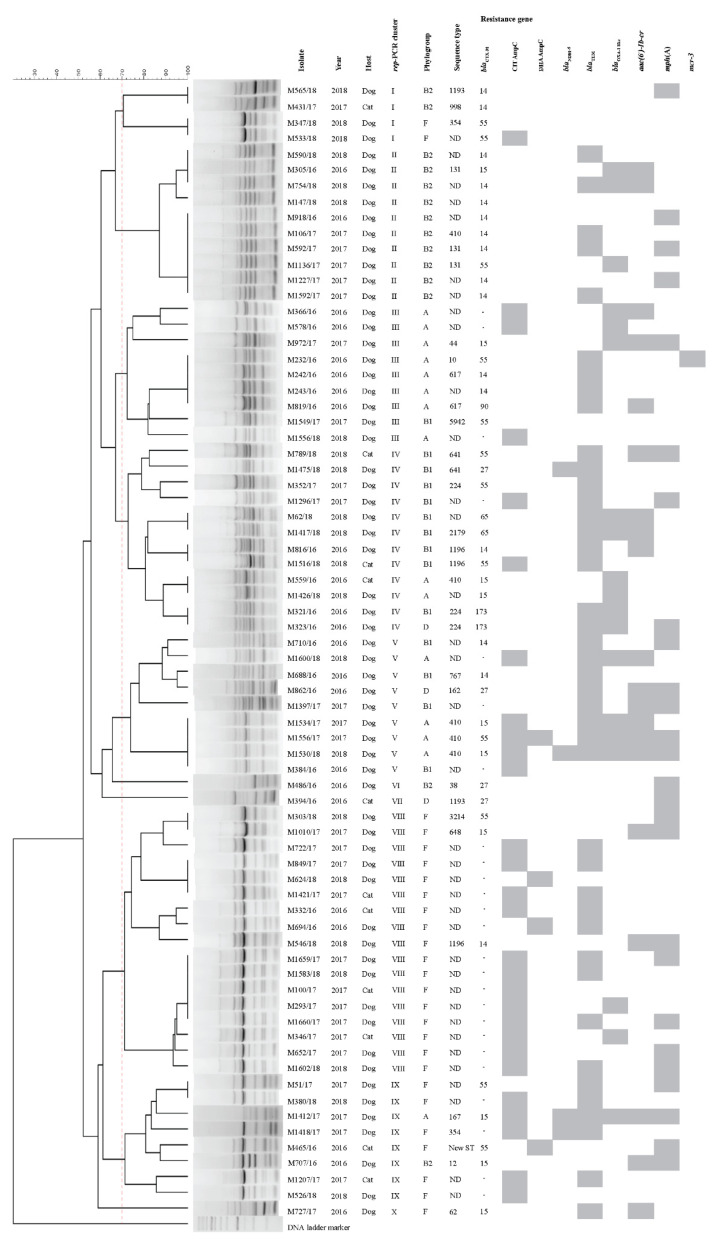
Antimicrobial resistance genes and genetic features based on repetitive extragenic palindromic sequence-PCR (*rep*-PCR), phylogroup and sequence type of 60 canine and 12 feline extended-spectrum cephalosporin-resistant uropathogenic *Escherichia coli* in Thailand, 2016–2018.

Based on the Clermont phylo-typing scheme, the UPEC isolates belonged to five phylogroups, namely, F (36.11%, 26/72), A (20.83%, 15/72), B1 (19.44%, 14/72), B2 (19.44%, 14/72) and D (4.17%, 3/72). Ten repetitive extragenic palindromic sequence-PCR (*rep*-PCR) clusters (cluster I to X) were classified by more than a 70% similarity of *rep*-PCR fingerprint patterns [17]. All of the 17 isolates in cluster VIII and the 10 isolates in cluster II were consistently membered in phylogroup F and phylogroup B2, respectively. Clusters III and IV were composed of isolates in phylogroups A (eight isolates) and B1 (nine isolates), respectively. Other clusters were heterogeneously associated with different phylogroups (Figure 1).

The MLST analysis of 31 representative UPEC isolates, which were selected based on different characteristics (*bla*_CTX-M_ type, phylogroup and *rep*-PCR cluster), four *bla*_NDM-5_-positive isolates and one *mcr-3*-positive isolate resulted in 21 previously assigned STs and one new allelic profile (101-88-97-29-26-79-2) (Figure 1). The *bla*_CTX-M_ variants could be detected in multiple STs, and different *bla*_CTX-M_ variants could be found in each isolate in the same ST. Up to nine STs, including ST10, ST131, ST224, ST354, ST410, ST641, ST1196, ST3214 and ST5942, contained *bla*_CTX-M-55_; *bla*_CTX-M-14_ was found in seven STs (ST131, ST410, ST617, ST767, ST998, ST1193 and ST118). The *bla*_CTX-M-15_ was also detected in seven STs (ST12, ST44, ST62, ST131, ST167, ST410 and ST64), and *bla*_CTX-M-27_ was found in ST162, ST641 and ST1193. The *bla*_CTX-M-65_, *bla*_CTX-M-90_ and *bla*_CTX-M-173_ were harboured by ST2179, ST617 and ST224, respectively. The four *bla*_NDM-5_-carrying isolates were ST167 (phylogroup A), ST354 (phylogroup F), ST410 (phylogroup A) and ST641 (phylogroup B1), and the ST354 isolate was *bla*_CTX-M_-negative. The *mcr-3*-positive isolate was ST10 containing *bla*_CTX-M-55_.

## 3. Discussion

This study showed the heterogenicity of ESC-resistant UPEC isolated from canine and feline clinical specimens in terms of MDR, virulence gene carriage and genetic background. Various ESBL- and pAmpC-encoding genes, including *bla*_CTX-M_ variants and CIT and DHA AmpC groups, were prevalent in these isolates, and *bla*_NDM-5_ was first detected in animal sources in Thailand. All the detected *bla*_CTX-M_ variants belonging to group 1 (*bla*_CTX-M-15_, *bla*_CTX-M-55_ and *bla*_CTX-M-173_) or group 9 (*bla*_CTX-M-14_, *bla*_CTX-M-27_, *bla*_CTX-M-65_ and *bla*_CTX-M-90_) are the predominant ESBL genes in *E. coli* from human clinical specimens in Thailand [14]. In this study, *bla*_CTX-M-14_ was the most prevalent ESBL gene, followed by *bla*_CTX-M-55_ and *bla*_CTX-M-15_. Here, *bla*_CTX-M-55_ was first characterised in Thailand, and four major variants, including *bla*_CTX-M-14, -15, -27_ and _-55_, which also widely disseminate in ESC-resistant *E. coli* from human sources [18]. In companion animals, *bla*_CTX-M-14_ is mostly carried by feline UPEC [19], and *bla*_CTX-M-15_ and *bla*_CTX-M-55_ are the most frequently occurring variants found across the globe, especially *bla*_CTX-M-55_ in Asian countries [5,11,20,21,22,23,24,25,26]. Similar studies from Portugal identified faecal ESC-resistant *E. coli* containing several *bla*_CTX-M_ variants in healthy and sicked dogs and cats that might be a potential source for zoonotic transmission [27,28]. We provide further evidence that the widespread *bla*_CTX-M_ genes were shared among *E. coli* from human and animal origins. Besides *bla*_CTX-M_, the CIT and DHA groups of pAmpC and *bla*_TEM_ were found in half of the studied isolates. The *bla*_CMY-2_, which is a major CIT member, is more prevalent than *bla*_CTX-M_, which disseminates among ESC-resistant *E. coli* from canine stools in South Korea [11], but pAmpC and other *bla* genes were not specifically identified by sequencing in this study. Due to the presence of various classes of β-lactamase-encoding genes, the effectiveness of different β-lactam generations, which is the widest antibiotic group used in veterinary and human medicine, is limited by the accumulation and development of resistance.

The canine and feline UPEC populations carried plasmid-borne antimicrobial resistance genes, such as *aac(6′)-Ib-cr*, *mph*(A), *bla*_NDM-5_ and *mcr-3*, promoting additional resistance to higher generations and different groups of antimicrobials. In addition to stepwise mutations of gyrase and topoisomerase genes, *aac(6′)-Ib-cr* is usually co-harboured in fluoroquinolone and ESC-resistant *E. coli* [15,29], which is of concern due to the horizontal spread among Gram-negative pathogens. To our knowledge, this is the first report of carbapenemase *bla*_NDM-5_ and plasmid-mediated colistin resistance *mcr-3* in *E. coli* from companion animals in Thailand. The *bla*_NDM-5_ is a frequent variant carried by high-risk human-associated ExPEC clones [30,31], and isolation from animal sources has been increasingly reported worldwide [32]. These alarm a crisis of resistance to last-resort antimicrobials in veterinary medicine. With the emergence of MDR, carbapenems are introduced for extra label treatment of serious Gram-negative infections in small animal practices, whereas colistin is not available because of the lack of recommendation from pharmacokinetic data in dogs and cats. The increased spread of resistance is possible if carbapenem is continuously being used without strict regulations in small animal practices. As colistin is not used in companion animals, livestock or humans are probable sources of *mcr-3*. The *mcr-3* gene from Enterobacteriaceae has been also reported from Thai people and swine, suggesting a common prevalent type shared among different hosts in Thailand [6,33]. Companion animals living in complex proximities to humans, animals and food are prone to acquiring a variety of resistance genes and microorganisms [22].

Various clones and genetic linkage of the UPEC isolates have been illustrated by STs, phylogroups and DNA fingerprints. They belonged to phylogroups A, B1, B2, D and F, of which the latter is the most common phylogroup, followed by phylogroup A in our study. Recent studies found the intestinal carriage of the phylogroups A, B1, B2 and D to be associated with ESBL and pAmpC production in healthy and diseased dogs and cats [27,28]. Previous evidence supports the assumption that uropathotypes are mainly members of the virulent phylogroup B2, and the commensal isolates are phylogroup A [12]. An Australian study also found that phylogroups A and B1 were dominant among canine and feline fluoroquinolone-resistant *E. coli*, causing extraintestinal infections [15]. Clonal expansion and genetic relatedness could be deduced using DNA fingerprint cluster analysis, demonstrating an association of the major clusters II, III, IV and VIII with the phylogroups B2, A, B1 and F, respectively. A variety of virulence genes associated with uropathogenesis were found in the UPEC isolates and in all the phylogroups identified. However, the isolates in phylogroup B2, theoretically, carried the highest numbers of virulence genes. Based on genetic and virulence characteristics and clinical sources, our results support the uropathogenic potential of non-B2 *E. coli* phylogroups in canine and feline urinary tract infections.

We identified multiple high-risk, human-associated ExPEC STs with a significant public health implication, such as ST410-F (ST-phylogroup), ST131-B2, ST354-F, ST648-F and ST38-D, [34]. Of these, ST38, ST131 and ST410 are the major STs of ESBL-producing *E. coli* causing human infections in Thailand [14]. The frequently identified high-risk STs from canine and feline clinical specimens are ST38, ST131, ST224, ST354, ST648 and ST2179 [15,35]. Interspecies transmission and clonal dissemination are presumable by shared STs and the high similarity of genetic characteristics of strains from different sources [36]. The global spread of *bla*_CTX-M-15_-carrying ST131, which mainly belongs to the virulent phylogroup B2, is considerably worrying due to its MDR and virulent traits associated with UTIs and fatal outcomes in humans [37]. The expansion of ST131 in small animals has been increasingly proposed, emphasising its clinical significance [19,20]. Shared high-risk clones between pets and humans in the same environment are a public health and occupational risk for pet-associated people [38]. Indeed, the identification and virulence determinants of human-associated ExPEC clones from diseased dogs and cats in this study confirmed the pathogenic potentials in animals, and infected pets or carriers may be a source of reverse transmission to humans. Environmental contamination in veterinary settings and colonisation in personnel should be further monitored to improve preventive measures against infection in animals.

Companion animals could be a potential niche for multiple high-risk ExPEC clonal expansion in the community. Clonally related *bla*_CTX-M_-carrying ST410 is spread among human and veterinary clinical settings, livestock and wild avian species, but the transmission scenarios cannot be clearly clarified [36,39]. Dogs and cats play a role as a reservoir of ST648 harbouring *bla*_CTX-M_ group 1, which is sporadically found in humans [35,40,41,42]. An accumulation of multiple β-lactamase-encoding genes is more common in the high-risk ExPEC lineages. One study has reported an abundant co-harbouring of *bla*_CTX-M-15_, *bla*_TEM-1_, *bla*_OXA-1_, *bla*_CMY-2_ and *aac(6*′)-*Ib-cr* in ESC-resistant ST410 strains from infected animals [20]. Besides *bla*_CTX-M-15_, the most prevalent *bla*_CTX-M-14_ and *bla*_CTX-M-55_ were carried by ST131 isolates in our study. Moreover, ST167, ST354, ST410 and ST641 carrying *bla*_NDM-5_ found in this study confirm the capability to evolve by additional gene acquisition in these lineages. Nearly identical resistance genotypes with an additional *bla*_NDM-5_ co-carriage in the proliferative high-risk clones imply a more critical situation of antimicrobial resistance to limit options and reduce the effectiveness of last-line antimicrobials in small animal practices in Thailand.

The various plasmid replicons identified in the MDR UPEC isolates support the assumption that the plasmids could play a role in resistance and virulence contribution. The number of replicons identified in each isolate may not relate to the number of the carried plasmids. Closely related multi-replicon plasmids containing resistance and virulence genes in *E. coli* from human and dogs have been characterised by whole genome sequencing (WGS) [7,30]. However, transmissibility was not assessed in this study. Virulence and antimicrobial resistance genes may be co-harboured for the co-selection of the resistant and virulent traits, and plasmid recombination can enhance higher pathogenicity and create hybrid *E. coli* pathotypes [43]. Resistance determinants may be alternatively integrated in the plastic bacterial chromosome, which is supported by variable chromosomal regions or plasmids for *bla*_CTX-M-15_ localisation in ExPEC ST410 [39]. The *bla*_CTX-M-14_, *bla*_CTX-M-15_, *bla*_CTX-M-27_ and *bla*_CTX-M-55_, found in multiple STs, support the active mobility of the elements for spreading among ExPEC from human and animal origins. Further analyses of gene localisation and the characterisation of mobile genetic elements can increase our understanding of the modes of transmission and co-selection of the resistance elements and strains and are useful for monitoring and creating interventions to limit the spread.

The *bla*_NDM-5_ in ST167, ST354, ST410 and ST641 supports the evolution of resistance, which is more successful in extensively drug-resistant ExPEC populations. In a previous study, WGS analysis was used to identify *bla*_NDM-5_ localisation on the IncFII plasmid in ST167 from dogs and cats in the USA [29]. A narrow host range of IncX3 plasmid is a vehicle for *bla*_NDM-5_ spread in ST167 in China [31], but this IncX3 was not detected in our carbapenem-resistant isolates. All the *bla*_NDM-5_-carrying isolates were *intI*1-positive. The integron collecting *bla*_NDM-5_ and other resistance elements associated with plasmids have been characterised [7]. Although *mcr* and *bla*_NDM_ co-carriage was not detected in this study, co-harbouring *bla*_NDM-5_ and *mcr-1* in ST167 on different plasmids has been reported in a hospitalised patient with pneumonia in Japan [44]. Potential development is likely by gene acquisition when the strains will be introduced in the environment with antimicrobial exposure. Carbapenems are extra-labelly prescribed in animal cases with life-threatening MDR bacterial infections in Thailand; however, the history of carbapenem administration in dogs was not assessed. The NDM-5-producing ExPEC have emerged in hospitalised companion animals without carbapenem treatment within a 1-month period in the USA [29]. Nevertheless, antimicrobial use is a key factor contributing to resistance selection and spread. Due to the emergence of carbapenem and colistin resistance, the selection of these drugs, which are considered last-line antimicrobials in human medicine, should be avoided if the causative bacteria are still susceptible to available drugs for veterinary purposes.

The urinary tract is a body site that is mostly affected by ExPEC infection. Almost half of the *E. coli* (151/344) in our collection were from a UTI. It is likely that UPEC were indigenous *E. coli* in the intestinal tract, which can ascendingly be translocated through the defective urinary pathway. Both ESC and amoxicillin/clavulanic acid exposure promotes the intestinal carriage of ESBL-producing Enterobacteriaceae, which can be a source of extraintestinal infections [32]. Huang et al. found that 22.8% (65/283) of *E. coli* from companion animal specimens produce ESBLs, and more than 70% of those derive from urine, pus in the uterus and wounds [21]. In the present study, a high proportion (43.06%) of the ESC-resistant UPEC expressed the ESBL phenotype by CDT, but the expression in the remaining *bla*_CTX-M_-positive isolates might be masked by other co-existing genes, such as AmpC cephalosporinase, carbapenemase or others. Thus, we recommend including various surrogate cephalosporin generations, amoxicillin/clavulanic acid and carbapenems, together with ESBL phenotype detection in β-lactam panels, for antimicrobial susceptibility testing for surveillance purposes. The MDR of the ESC-resistant UPEC strains negatively impacted antimicrobial uses in small animal practices, especially the empirical selection of first- and second-line antimicrobials. To encourage diagnostic and antimicrobial stewardships, AST panels customising the drugs used for UTIs are strongly recommended for effective treatment. With a low nitrofurantoin resistance rate, this drug could still be an effective choice. However, AST is strongly recommended for the most reasonably appropriate treatment. This study focused on only UPEC, which is a subtype of ExPEC, from dogs and cats. In addition, ExPEC is a major pathogen associated with other extraintestinal diseases in small animals, such as pneumonia, endometritis and prostatitis [29,45,46], but the samples were difficult to obtain for routine clinical diagnosis because of the invasive sample collection procedures. Further studies on the virulence and genetic background of ExPEC in companion animals of other clinical relevance may help to understand the pathogenesis of ExPEC in small animals and the epidemiological linkage with strains from humans. Shedding in the environment through faecal excretion is also an important indirect mode of transmission, especially in animal healthcare settings where antimicrobials are intensively consumed. Control and prevention of spread should be undertaken in veterinary hospitals by prescription restriction, strict hygiene and decontamination. The development of resistance and spread can be impeded, and the effectiveness of antimicrobials can be prolonged by the implementation of antimicrobial stewardship programs in veterinary practices.

## 4. Materials and Methods

### 4.1. Bacterial Isolates, Species Identification and DNA Isolation

The *E. coli* isolates were obtained from clinical specimens submitted to the Veterinary Diagnostic Laboratory of the Faculty of Veterinary Science, Chulalongkorn University, between 2016 and 2018. The criteria used for selection were *E. coli* presenting resistance to 3GCs that were isolated from canine and feline samples associated with UTIs. Species and 3GC resistance were initially identified by GN-ID and AST-GN65 of the Vitek^®^ automated system in routine diagnostic activity (bioMerieux, Marcy l’Etoile, France). Bacteria were preserved in tryptic soy broth with 20% glycerol stock and kept at −80 ℃. Species were confirmed using matrix-assisted laser desorption and ionisation time-of-flight mass spectrometry (MALDI-TOF MS) (Microflex^®^ Biotyper) (Bruker, Dalnotik, Bremen, Germany), and genomic DNA was extracted by using the Nucleospin Tissue DNA extraction kit (Macherey-Nagel, Düren, Germany) from pure cultures after recovery on 5% sheep blood agar. The DNA was stored at −20 ℃ to be used as DNA template for PCR-based assays.

### 4.2. Antimicrobial Susceptibility Testing

The ESBL phenotypic confirmation test was conducted using a CDT [47]. The antimicrobial susceptibility profile was determined by using customised Sensititre COMPAN1F and EUVSEC 96-well plates (Trek Diagnostic Systems, East Grinstead, West Sussex, UK), based on the broth microdilution technique [47]. Nitrofurantoin susceptibility was obtained from routine diagnostic results using the Vitek^®^ system. To interpret as susceptible (S), intermediate (I) or resistant (R), veterinary-specific breakpoints (µg/mL) from the Clinical and Laboratory Standard Laboratory Institute (CLSI) were used for cefazolin (≥32), cefovecin (≥8), ceftiofur (≥8), cefpodoxime (≥8), enrofloxacin (≥4), marbofloxacin (≥4), gentamicin (≥8) and amikacin (≥8) [48]. The CLSI clinical breakpoints for humans were used for ampicillin (≥32), amoxicillin/clavulanic acid (≥32/16), cefotaxime (≥4), cefoxitin (≥32), meropenem (≥4), azithromycin (>32), nalidixic acid (≥32), ciprofloxacin (≥1), tetracycline (≥16), doxycycline (≥16), sulfamethoxazole (≥512), trimethoprim (≥16), sulfamethoxazole/trimethoprim (76/4), chloramphenicol (≥32), nitrofurantoin (≥128) and colistin (≥4) [47]. The resistance breakpoints (mg/L) of ticarcillin (>16), ticarcillin/clavulanic acid (>16/2) and tigecycline (>0.5) were referred to those specified by the European Committee for Antimicrobial Susceptibility Testing (EUCAST) [49].

### 4.3. Detection of Antimicrobial Resistance Genes

The *bla*_TEM_, *bla*_SHV_, *bla*_OXA-1-like_ and *bla*_CTX-M_ (group 1, 2 and 9) genes were detected via M-PCRs, and the *bla*_CTX-M_ group 8/25 was detected using a single PCR [50,51]. The presence of plasmid-mediated AmpC β-lactamase genes, including ACC, FOX, MOX, DHA, CIT and EBC groups, was examined via M-PCR [50]. The carbapenem-resistant isolates were included for M-PCRs for the detection of carbapenemase-encoding genes [52]. PMQR genes, including *aac(6′)-Ib-cr*, *qnrA*, *qnrB*, *qnrC*, *qnrD*, *qnrS* and *qepA*, and a plasmid-mediated azithromycin resistance gene *mph*(A) were detected using PCRs [53,54,55,56,57,58,59]. The *mcr-1 to -9* genes were screened in the colistin-resistant isolates using M-PCRs [60,61]. The *bla*_CTX-M_, *bla*_NDM_ and *mcr* variants were identified using nucleotide sequencing. All PCR reactions were carried out in 25 μL of reaction mixture including 5X Firepol^®^ Master Mix Ready-to-Load (Solis Biodyne, Tartu, Estonia), 0.2 µM of each primer and 1 μL of DNA template. The primers used for the detection of antimicrobial resistance genes are listed in Supplementary Table S2.

### 4.4. Detection of ExPEC Virulence Genes

The ExPEC virulence genes associated with uropathogenesis that encode adhesins (*iha*, *papC*, *crl*, *sfa/foc* and *afa/dra)*, cytotoxins (*hlyA*, *cnf1/2*, *sat* and *vat)* and the iron acquisition system (*iucD* and *iroN*) were detected using M-PCRs [62]. The presence of *fimH* was identified using a single specific PCR [63]. The PCR mixture was composed of reagents as described above.

### 4.5. Plasmid Replicon Typing and Integrase Detection

Plasmid replicons were investigated using five M-PCRs, including multiplex 1 for HI1, HI2 and I1-Iγ, multiplex 2 for X, L/M and N, multiplex 3 for FIA, FIB and W, multiplex 4 for Y, P and FIC and multiplex 5 for A/C, T and FIIAs, as well as three simplex PCRs for F, K and B/O [64]; *intI*1 was detected using a specific PCR [65].

### 4.6. Molecular Typing

The *E. coli* phylogroups including A, B1, B2, C, D, E, F and *Escherichia* cryptic clade I were characterised by the Clermont phylo-typing scheme [66]. Genetic relatedness was illustrated by a dendrogram construction based on *rep*-PCR fingerprint analysis. The repetitive regions were amplified by 1 µM REP-1 primer (5′-GCGCCGICATCAGGC-3′) and 1 µM REP-2 primer (5′-ACGTCTTATCAGGCCTAC-3′) in 50 µL of PCR reaction mixture [67]. The DNA fingerprint patterns were illustrated by 1.5% agarose gel electrophoresis, and the dendrogram was constructed using the Bionumeric software (Applied Maths, Sint-Martens-Latem, Belgium) with 1% position tolerance. Isolates presenting more than 70% similarity were categorised in the same cluster. Representative isolates of identical phylogroups and *rep*-PCR patterns that contained each *bla*_CTX-M_ variants and the strains having *bla*_NDM-5_ or *mcr-3* genes were selected for MLST. The STs were identified by the Achtmann MLST scheme by comparing them to sequences and alleic profiles on the Enterobase website (https://enterobase.warwick.ac.uk/species/ecoli/) (accessed on 15 March 2021) [13].

## 5. Conclusions

This study highlights that canine and feline ESC-resistant UPEC are genetically linked to high-risk, MDR, human-associated ExPEC clones. Various genes corresponding resistance to critically and highly critically important antimicrobials, including *bla*_CTX-M_ types, pAmpC, *bla*_NDM-5_ and *mcr-3*, were first detected in *E. coli* from small animal origins in Thailand. This evidence supports the emergence of resistance to last-resort antimicrobials in companion animals, highlighting a “spillover” of the bacteria that may be bidirectionally transmitted between humans and pets.

## Data Availability

Data available from the authors upon reasonable request.

## Supplementary Materials

The following are available online at https://www.mdpi.com/article/10.3390/antibiotics10111374/s1, Table S1: Phylogroup, Origin, repetitive extragenic palindromic sequence-PCR (*rep*-PCR) cluster, integrase gene (*intI*1), plasmid replicon, virulence gene and antimicrobial resistance profiles of uropathogenic *Escherichia coli* isolated from dogs (60 isolates) and cats (12 isolates) in Thailand, 2016–2018., Table S2: List of primers for the detection of antimicrobial resistance genes.
