# Supplementary material for: Multiple and High-Risk Clones of Extended-Spectrum Cephalosporin-Resistant and blaNDM-5-Harbouring Uropathogenic Escherichia coli from Cats and Dogs in Thailand
Source: Antibiotics (Basel). 2021 Nov 10;10(11):1374. doi: 10.3390/antibiotics10111374 (PMC8614778; doi:10.3390/antibiotics10111374)
Supplement: Supplementary file 1 [file antibiotics-10-01374-s001.zip › antibiotics-1405064-supplementary.pdf]

Supplementary Materials

Table S1. Phylogroup, Origin, repetitive extragenic palindromic sequence-PCR (*rep*-PCR) cluster, integrase gene (*intI*1), plasmid replicon, virulence gene, and antimicrobial resistance phenotype and gene of uropathogenic *Escherichia coli* isolated from dogs (60 isolates) and cats (12 isolates) in Thailand between 2016 and 2018.

Phylogroup	Isolate No.	Year	Host	*rep*-PCR cluster	*intI*1	Plasmid Replicon	Virulence gene profile	Antimicrobial resistance gene	Antimicrobial resistance phenotype
A	M232/16	2016	Dog	III	+	F	*fimH*, *crl*, *iucD*	$bla_{\text{CTX-M-55}}$, bla_{TEM}, *mcr-3*	CDT-NAL-CIP-ENR-MAR-GEN-TET-DOX-TMP-SXT-CHL-COL
	M242/16	2016	Dog	III	+	F, FIB	*fimH*, *crl*, *iucD*, *sfa/foc*	$bla_{\text{CTX-M-14}}$, bla_{TEM}	CDT-NAL-CIP-ENR-MAR-GEN-TET-DOX-TMP-SXT-CHL
	M243/16	2016	Dog	III	+	F, FIB	*fimH*, *crl*, *iucD*, *sfa/foc*	$bla_{\text{CTX-M-14}}$, bla_{TEM}	CDT-NAL-CIP-ENR-MAR-GEN-TET-DOX-TMP-SXT-CHL
	M366/16	2016	Dog	III	+	F, FIA, FIB, I1-Iγ, N	*fimH*, *crl*, *iucD*	$bla_{\text{OXA-1-like}}$, CIT, *aac(6')-Ib-cr*	AMC-FOX-NAL-CIP-ENR-MAR-AMK-TET-DOX-TMP-SXT-CHL
	M559/16	2016	Cat	IV	-	F, FIA, FIB	*fimH*, *crl*, *iucD*	$bla_{\text{CTX-M-15}}$, $bla_{\text{OXA-1-like}}$	CDT-NAL-CIP-ENR-MAR-TET-DOX-TMP-SXT-CHL
	M578/16	2016	Dog	III	-	F, FIB	*fimH*, *crl*, *iroN*	$bla_{\text{OXA-1-like}}$, CIT	AMC- FOX-NAL-CIP-ENR-MAR-TET-DOX-TMP-SXT
	M819/16	2016	Dog	III	+	FIC	*fimH*, *crl*	$bla_{\text{CTX-M-90}}$, bla_{TEM}, *aac(6')-Ib-cr*	CDT-NAL-CIP-ENR-MAR-GEN-AMK-TET-DOX-AZI-TMP-SXT-CHL-COL
	M972/17	2017	Dog	III	+	F, FIA, FIB	*fimH*, *crl*, *iucD*, *sfa/foc*	$bla_{\text{CTX-M-15}}$, $bla_{\text{OXA-1-like}}$, *aac(6')-Ib-cr*, *mph*(A)	CDT-TIC- NAL-CIP-ENR-MAR-GEN-TET-DOX-AZI-TMP-SXT-CHL
	M1412/17	2017	Dog	IX	+	F, FIA, FIB	*fimH*, *crl*, *iucD*, *papC*, *sfa/foc*	$bla_{\text{CTX-M-15}}$, bla_{TEM}, $bla_{\text{OXA-1-like}}$, CIT, $bla_{\text{NDM-5}}$, *aac(6')-Ib-cr*, *mph*(A)	CDT-AMC-FOX-TIC-MER-IMP-NAL-CIP-ENR-MAR-GEN-AMK-TET-DOX-TMP-SXT-CHL
	M1534/17	2017	Dog	V	+	FIA, FIB	*fimH*, *crl*	$bla_{\text{CTX-M-15}}$, bla_{TEM}, $bla_{\text{OXA-1-like}}$, CIT, *aac(6')-Ib-cr*	AMC-FOX-NAL-CIP-ENR-MAR-TET-DOX-TMP-SXT-CHL-NFT
	M1556/17	2017	Dog	V	+	F, FIA, FIB	*fimH*, *crl*	$bla_{\text{CTX-M-55}}$, bla_{TEM}, $bla_{\text{OXA-1-like}}$, CIT, DHA, *aac(6')-Ib-cr*, *mph*(A)	AMC-FOX-TIC-NAL-CIP-ENR-MAR-GEN-TET-DOX-AZI-TMP-SXT
	M1426/18	2018	Dog	IV	-	F, FIA, FIB, Y	*fimH*, *crl*, *iucD*	$bla_{\text{CTX-M-15}}$, $bla_{\text{OXA-1-like}}$	CDT-AMC-TIC-NAL-CIP-ENR-MAR-GEN-AMK-TET-DOX-TMP-SXT-CHL

	M1530/18	2018	Dog	V	+	FIA, FIB	*fimH*, *crl*	$bla_{\text{CTX-M-15}}$, bla_{TEM}, $bla_{\text{OXA-1-like,}}$ CIT, $bla_{\text{NDM-5}}$ *aac(6′)-Ib-cr*, *mph*(A)	AMC-FOX-TIC-MER-IMP-NAL-CIP-ENR-MAR-GEN-TET-DOX-AZI-TMP-SXT-CHL-NFT
	M1556/18	2018	Dog	III	-	F, FIA, FIB	*fimH*, *crl*, *iucD*	CIT	CDT-AMC-FOX-TIC-NAL-CIP-ENR-MAR-GEN-TET-DOX-TMP-SXT-NFT
	M1600/18	2018	Dog	V	+	FIA, FIB	*fimH*, *crl*	bla_{TEM}, $bla_{\text{OXA-1-like}}$, CIT, *aac(6′)-Ib-cr*	AMC-FOX-TIC-NAL-CIP-ENR-MAR-GEN-TET-DOX-TMP-SXT-CHL-NFT
B1	M321/16	2016	Dog	IV	+	-	*fimH*, *crl*, *cnf1/2*	$bla_{\text{CTX-M-173}}$, bla_{TEM}, $bla_{\text{OXA-1-like}}$	CDT-NAL-CIP-ENR-MAR-GEN-TET-DOX-TMP-SXT-CHL
	M384/16	2016	Dog	V	+	F, FIB, I1-Iγ, N	*fimH*, *crl*, *iucD*, *iroN*	CIT	AMC-FOX-TET-DOX-CHL-NFT
	M688/16	2016	Dog	V	+	F, FIB, I1-Iγ	*fimH*, *crl*, *iucD*, *iroN*	$bla_{\text{CTX-M-14}}$, bla_{TEM}	CDT-GEN-TET-DOX-TMP-SXT-CHL-NFT
	M710/16	2016	Dog	V	+	F, FIB	*fimH*, *crl*, *iucD*, *iroN*	$bla_{\text{CTX-M-14}}$, bla_{TEM}, *mph*(A)	CDT-NAL-GEN-TET-DOX-TMP-SXT-CHL
	M816/16	2016	Dog	IV	+	I1-Iγ, FIC	*fimH*, *crl*	$bla_{\text{CTX-M-14}}$, bla_{TEM}, *aac(6′)-Ib-cr*	CDT-NAL-CIP-ENR-MAR-GEN-AMK-TET-DOX-AZI-TMP-SXT-CHL-COL
	M352/17	2017	Dog	IV	+	I1-Iγ	*fimH*, *crl*	$bla_{\text{CTX-M-55}}$, bla_{TEM}	CDT-NAL-CIP-ENR-MAR-GEN-AMK-TET-DOX-TMP-SXT-CHL
	M1296/17	2017	Dog	IV	-	F, I1-Iγ	*fimH*, *crl*	bla_{TEM}, CIT, *mph*(A)	AMC-FOX-TIC-NAL-CIP-ENR-MAR-GEN-AMK-TET-DOX
	M1397/17	2017	Dog	V	-	FIA, FIB	*fimH*, *crl*	bla_{TEM}, *aac(6′)-Ib-cr*, *mph*(A)	AMC-FOX-TIC-NAL-CIP-ENR-MAR-GEN-AMK-TET-DOX-AZI-TMP-SXT
	M1549/17	2017	Dog	III	-	F, FIB, N	*fimH*, *crl*, *iucD*, *papC*, *cnf1/2*, *hlyA*, *sat*, *iha*	$bla_{\text{CTX-M-55}}$	CDT-NAL-CIP-ENR-MAR-GEN-TET-DOX-CHL-NFT
	M62/18	2018	Dog	IV	+	F, FIB	*fimH*, *crl*, *iucD*, *papC*, *cnf1/2*, *hlyA*, *sat*, *iha*	$bla_{\text{CTX-M-65}}$, , bla_{TEM}, $bla_{\text{OXA-1-like}}$, *aac(6′)-Ib-cr*	CDT-NAL-CIP-ENR-MAR-AMK-TET-DOX-TMP-SXT-CHL
	M789/18	2018	Cat	IV	-	F, FIB, N	*fimH*, *crl*, *iucD*, *papC*, *cnf1/2*, *hlyA*, *sat*, *iha*	$bla_{\text{CTX-M-55}}$, bla_{TEM}, *aac(6′)-Ib-cr*, *mph*(A)	CDT-NAL-CIP-ENR-MAR-AMK-TET-DOX-AZI-CHL
	M1417/18	2018	Dog	IV	-	F, FIB	*fimH*, *crl*, *iucD*, *iroN*	$bla_{\text{CTX-M-65}}$, bla_{TEM}, $bla_{\text{OXA-1-like}}$, *aac(6′)-Ib-cr*	CDT-NAL-CIP-ENR-MAR-TET-DOX-TMP-SXT-CHL
	M1475/18	2018	Dog	IV	+	F, I1-Iγ	*fimH*, *crl*, *hlyA*	$bla_{\text{CTX-M-27}}$, bla_{TEM}, $bla_{\text{NDM-5}}$	AMC-FOX-TIC-MER-IMP-NAL-CIP-ENR-MAR-GEN-AMK-TET-DOX-AZI-TMP-SXT-CHL-NFT

	M1516/18	2018	Cat	IV	-	F, N	*fimH, crl,*	*bla*$_{CTX-M-55}$, *bla*$_{TEM}$, CIT	CDT-AMC-FOX-TIC-NAL-CIP-ENR-MAR-GEN-TET-DOX-TMP-SXT-CHL
B2	M305/16	2016	Dog	II	-	F, FIA, FIB	*fimH, iucD, papC, cnf1/2, hlyA, sat, iha*	*bla*$_{CTX-M-15}$, *bla*$_{OXA-1-like}$, *aac(6′)-Ib-cr*	CDT-NAL-CIP-ENR-MAR-GEN-AMK
	M486/16	2016	Dog	VI	-	FIA, FIB	*fimH, iucD*	*bla*$_{CTX-M-27}$, *mph*(A)	CDT-NAL-CIP-ENR-MAR-TET-DOX-AZI-TMP-SXT
	M707/16	2016	Dog	IX	+	-	*fimH, crl, papC, cnf1/2, iroN*	*bla*$_{CTX-M-15}$, *aac(6′)-Ib-cr*, *mph*(A)	CDT-FOX-TIC-CIP-ENR-MAR-GEN-TET-AZI-TMP-SXT-CHL
	M918/16	2016	Dog	II	-	F, FIA, FIB	*fimH, crl, iucD, sat, iha*	*bla*$_{CTX-M-14}$, *mph*(A)	CDT-NAL-CIP-ENR-MAR-GEN-AZI-COL
	M106/17	2017	Dog	II	-	F, FIA, FIB	*fimH, crl, cnf1/2, hlyA, sat, iha, afa/dra*	*bla*$_{CTX-M-14}$, *bla*$_{TEM}$	CDT-NAL-CIP-ENR-GEN-AMK-TET-DOX-SXT
	M431/17	2017	Cat	I	-	I1-Iγ	*fimH, crl, cnf1/2, sfa/foc, hlyA, iroN*	*bla*$_{CTX-M-14}$	CDT-NAL-ENR
	M592/17	2017	Dog	II	+	F, FIA, FIB, I1-Iγ	*fimH, crl, iucD, papC, cnf1/2, hlyA, sat, iha*	*bla*$_{CTX-M-14}$, *bla*$_{TEM,}$ *mph*(A)	CDT-TIC-NAL-CIP-ENR-MAR-GEN-TET-DOX-CHL
	M1136/17	2017	Dog	II	-	F, FIA, FIB	*fimH, crl, iucD, papC, cnf1/2, sat, iha*	*bla*$_{CTX-M-55}$, *bla*$_{OXA-1-like}$, *aac(6′)-Ib-cr*	CDT-NAL-CIP-ENR-MAR-GEN-AMK-TET-DOX
	M1227/17	2017	Dog	II	+	F, FIA, FIB, I1-Iγ	*fimH, crl, iucD, afa/dra*	*bla*$_{CTX-M-14}$, *bla*$_{TEM}$, CIT, *mph*(A)	CDT-AMC-FOX-TIC-NAL-CIP-ENR-MAR-GEN-TET-DOX-AZI-TMP-SXT
	M1592/17	2017	Dog	II	-	F, FIA, FIB	*fimH, crl, iucD, iroN*	*bla*$_{CTX-M-14}$, *bla*$_{TEM}$	CDT-TIC-NAL-CIP-ENR-MAR-GEN-TET-DOX-CHL
	M147/18	2018	Cat	II	-	FIA, FIB	*fimH, crl, iucD*	*bla*$_{CTX-M-14}$	CDT-NAL-CIP-ENR-MAR-DOX-TMP-SXT
	M565/18	2018	Dog	I	-	F, FIA, FIB	*fimH, crl, iucD, papC, cnf1/2, hlyA, sat, iha*	*bla*$_{CTX-M-14}$, *mph*(A)	CDT-NAL-CIP-ENR-MAR-TET-DOX-AZI-TMP
	M590/18	2018	Dog	II	-	F, FIA, FIB	*fimH, crl*	*bla*$_{CTX-M-14}$, *bla*$_{TEM}$	CDT-AMC-FOX-TIC-NAL-CIP-ENR-MAR-GEN-TET-DOX-AZI-CHL
	M754/18	2018	Dog	II	-	F, FIA, FIB	*fimH, crl, afa/dra*	*bla*$_{CTX-M-14}$, *bla*$_{TEM}$, *bla*$_{OXA-1-like}$, *aac(6′)-Ib-cr*	CDT-NAL-CIP-ENR-MAR-AMK
D	M323/16	2016	Dog	IV	+	F, I1-Iγ	*fimH, crl,*	*bla*$_{CTX-M-173}$, *bla*$_{TEM}$, *bla*$_{OXA-1-like}$, *mph*(A)	CDT-NAL-CIP-ENR-MAR-GEN-TET-DOX-TMP-SXT-CHL
	M394/16	2016	Cat	VII	-	F, FIA, FIB	*fimH, crl, iucD, sfa/foc, iha*	*bla*$_{CTX-M-27}$, *mph*(A)	CDT-NAL-CIP-ENR-MAR-TET-DOX-AZI-TMP-SXT

	M862/16	2016	Dog	V	+	F, FIB	*fimH*, *crl*, *iucD*	*bla*CTX-M-27, *bla*TEM, *aac(6′)-Ib-cr*, *mph*(A)	CDT-NAL-CIP-ENR-MAR-GEN-AMK-TET-DOX-AZI-TMP-SXT-CHL-COL
F	M332/16	2016	Cat	VIII	+	F, FIA, FIB, A/C	*fimH*, *crl*, *sfa/foc*	*bla*TEM, CIT	AMC-FOX-TMP-SXT-CHL
	M465/16	2016	Cat	IX	+	F, FIB, I1-Iγ	*fimH*, *crl*, *iucD*	*bla*CTX-M-55, DHA, *mph*(A)	CDT-AMC-FOX-NAL-CIP-ENR-MAR-GEN-TET-DOX-AZI-TMP-SXT-CHL
	M694/16	2016	Dog	VIII	-	F, FIB, I1-Iγ, N	*fimH*, *crl*	*bla*TEM, DHA	AMC-FOX-TIC-CIP-ENR-GEN-TET-DOX
	M727/16	2016	Dog	X	-	F, FIB, P	*fimH*, *crl*, *iucD*, *papC*, *sat*	*bla*CTX-M-15, *bla*TEM, *aac(6′)-Ib-cr*	CDT-NAL-TET-DOX-TMP-SXT
	M51/17	2017	Dog	IX	+	F, FIB, I1-Iγ	*fimH*, *crl*, *iucD*, *papC*	*bla*CTX-M-55, *bla*TEM, *mph*(A)	CDT-FOX-NAL-CIP-ENR-MAR-GEN-TET-DOX-AZI-TMP-SXT-CHL
	M100/17	2017	Cat	VIII	-	-	*fimH*, *crl*	CIT	AMC-FOX-NAL-CIP-ENR-MAR-TET-DOX-SXT-CHL
	M293/17	2017	Dog	VIII	+	F, FIA, FIB, A/C	*fimH*, *crl*, *sfa/foc*	*bla*OXA-1-like, CIT	AMC-FOX-TIC-NAL-CIP-ENR-MAR-GEN-TET-DOX-TMP-SXT-CHL-NFT
	M346/17	2017	Cat	VIII	+	F	*fimH*, *crl*, *papC*,	*bla*OXA-1-like, CIT	AMC-FOX-NAL-CIP-ENR-MAR-GEN-TET-DOX-TMP-SXT-CHL-NFT
	M652/17	2017	Dog	VIII	-	F, FIA, FIB	*fimH*, *crl*, *hlyA*	CIT, *mph*(A)	AMC-FOX-NAL-CIP-ENR-MAR-GEN-TET-DOX-AZI-TMP-SXT-CHL
	M722/17	2017	Dog	VIII	+	F, FIA, FIB, I1-Iγ	*fimH*, *crl*, *iucD*, *sfa/foc*	*bla*TEM, CIT	AMC-FOX-NAL-CIP-ENR-MAR-GEN-TET-DOX-TMP-SXT-CHL
	M849/17	2017	Dog	VIII	+	FIA, FIB, A/C	*fimH*, *crl*, *iucD*, *sfa/foc*	*bla*TEM, CIT	AMC-FOX-TIC-NAL-CIP-ENR-MAR-GEN
	M1010/17	2017	Dog	VIII	+	F, FIA, FIB	*fimH*, *crl*, *iucD*, *papC*, *cnf1/2*, *hlyA*	*bla*CTX-M-15, *bla*OXA-1-like, *aac(6′)-Ib-cr*, *mph*(A)	CDT-FOX-TIC-NAL-CIP-ENR-MAR-GEN-AMK-TET-DOX-AZI-TMP-SXT-CHL
	M1207/17	2017	Cat	IX	+	F, FIA, FIB	*fimH*, *crl*, *iucD*	*bla*TEM, CIT	AMC-FOX-TIC-NAL-CIP-ENR-MAR-GEN-AMK-TET-DOX-TMP-SXT
	M1418/17	2017	Dog	IX	+	F, FIA, FIB, I1-Iγ	*fimH*, *crl*, *iucD*	*bla*TEM, CIT, *bla*NDM-5	CDT-AMC-FOX-TIC-MER-IMP-NAL-CIP-ENR-MAR-GEN-AMK-TET-DOX-TMP-SXT
	M1421/17	2017	Cat	VIII	+	F, FIA, FIB, I1-Iγ	*fimH*, *crl*, *iucD*	*bla*TEM, CIT	AMC-FOX-TIC-NAL-CIP-ENR-MAR-GEN-AMK-TET-DOX-TMP-SXT-CHL

M1659/17	2017	Dog	VIII	-	F	*fimH*, *crl*, *papC*	*bla*TEM, CIT, *mph*(A)	AMC-FOX-TIC-NAL-CIP-ENR-MAR-TET-DOX-AZI-TMP-SXT-CHL
M1660/17	2017	Dog	VIII	-	F	*fimH*, *crl*, *papC*	*bla*TEM, CIT, *mph*(A)	AMC-FOX-NAL-CIP-ENR-MAR-TET-DOX-AZI-TMP-SXT-CHL
M303/18	2018	Dog	VIII	+	FIA, FIB	*fimH*, *crl*, *iucD*	*bla*CTX-M-55, *mph*(A)	CDT-NAL-CIP-ENR-MAR-GEN-TET-DOX-AZI-TMP-SXT-CHL
M347/18	2018	Dog	I	+	FIA, FIB	*fimH*, *crl*	*bla*CTX-M-55	CDT-FOX-NAL-CIP-ENR-MAR-GEN-TET-DOX-TMP-SXT-CHL-NFT
M380/18	2018	Dog	IX	+	F, FIA, A/C	*fimH*, *crl*, *iucD*, *sfa/foc*	*bla*TEM, CIT	AMC-FOX-TIC-NAL-CIP-ENR-MAR-GEN-TET-DOX-TMP-SXT-CHL
M526/18	2018	Dog	IX	+	F, I1-Iγ	*fimH*, *crl*	CIT	AMC-FOX-TIC-NAL-CIP-ENR-MAR-GEN-TET-DOX-CHL
M533/18	2018	Dog	I	+	F, I1-Iγ	*fimH*, *crl*, *iucD*	*bla*CTX-M-55, CIT	CDT-AMC-FOX-TIC-NAL-CIP-ENR-MAR-GEN-TET-DOX-AZI-CHL
M546/18	2018	Dog	VIII	+	F, FIA, FIB	*fimH*, *crl*, *iucD*, *sat*	*bla*CTX-M-14, *aac(6')-Ib-cr*, *mph*(A)	CDT-AMC-FOX-NAL-CIP-ENR-MAR-GEN-TET-DOX-AZI-TMP-SXT-CHL
M624/18	2018	Dog	VIII	-	F, FIB, I1-Iγ, A/C	*fimH*, *crl*	DHA	AMC-FOX-TIC-CIP-ENR-GEN-AMK-TET-DOX-AZI-CHL-COL
M1583/18	2018	Dog	VIII	-	F, Y	*fimH*, *crl*, *papC*	*bla*TEM, CIT	AMC-FOX-TIC-NAL-CIP-ENR-MAR-GEN-TET-DOX
M1602/18	2018	Dog	VIII	+	Y	*fimH*, *crl*	*bla*TEM, CIT, *mph*(A)	AMC-FOX-TIC-NAL-CIP-ENR-MAR-GEN-TET-DOX-AZI-TMP-SXT-CHL-NFT

+, indicate isolate that showed positive result; -, indicate isolate that showed negative result; CDT, Combination disk test; AMC, Amoxicillin/clavulanic acid; FOX, Cefoxitin; TIC, Ticarcillin/clavulanic acid; MER, Meropenem; IMP, Imipenem; NAL, Nalidixic acid; CIP, Ciprofloxacin; ENR, Enrofloxacin; MAR, Marbofloxacin; GEN, Gentamicin; AMK, Amikacin; TET, Tetracycline; DOX, Doxycycline; AZI, Azithromycin; TMP, Trimethoprim; SXT, Trimethoprim/sulfamethoxazole; CHL, Chloramphenicol; COL, Colistin; NFT, Nitrofurantoin.

Table S2. List of primers for the detection of antimicrobial resistance genes.

Primer name	Sequence (5′ –3′)	Gene target	Amplicon size (bp)	References
MultiTSO-T_for<br>MultiTSO-T_rev	CATTTCCGTGTCGCCCTTATTC<br>CGTTCATCCATAGTTGCCTGAC	TEM variants including TEM-1 and TEM-2	800	[1]
MultiTSO-S_for<br>MultiTSO-S_rev	AGCCGCTTGAGCAAATTAAAC<br>ATCCCGCAGATAAATCACCAC	SHV variants including SHV-1	713	[1]
MultiTSO-O_for<br>MultiTSO-O_rev	GGCACCAGATTCAACTTTCAAG<br>GACCCCAAGTTTCCTGTAAGTG	OXA-1, OXA-4 and OXA-30	564	[1]
MultiCTXMGp1_for<br>MultiCTXMGp1-2_rev	TTAGGAARTGTGCCGCTGYA<br>CGATATCGTTGGTGGTRCCAT	Variants of CTX-M group 1 including CTX-M-1, CTX-M-3 and CTX-M-15	688	[1]
MultiCTXMGp2_for<br>MultiCTXMGp1-2_rev	CGTTAACGGCACGATGAC<br>CGATATCGTTGGTGGTRCCAT	Variants of CTX-M group 2 including CTX-M-2	404	[1]
MultiCTXMGp9_for<br>MultiCTXMGp9_for	TCAAGCCTGCCGATCTGGT<br>TGATTCTCGCCGCTGAAG	Variants of CTX-M group 9 including CTX-M-9 and CTX-M-14	561	[1]
CTX-Mg8/25_for<br>CTX-Mg8/25_rev	AACRCRCAGACGCTCTAC<br>TCGAGCCGGAASGTGTYAT	CTX-M-8, CTX-M-25, CTX-M-26 and CTX-M-39 to CTX-M-41	326	[1]
MultiCaseACC_for<br>MultiCaseACC_rev	CACCTCCAGCGACTTGTTAC<br>GTTAGCCAGCATCACGATCC	ACC-1 and ACC-2	346	[1]
MultiCaseFOX_for<br>MultiCaseFOX_rev	CTACAGTGCGGGTGGTTT<br>CTATTTGCGGCCAGGTGA	FOX-1 to FOX-5	162	[1]
MultiCaseMOX_for<br>MultiCaseMOX_rev	GCAACAACGACAATCCATCCT<br>GGGATAGGCGTAACTCTCCCAA	MOX-1, MOX-2, CMY-1, CMY-8 to CMY-11 and CMY-19	895	[1]
MultiCaseDHA_for<br>MultiCaseDHA_rev	TGATGGCACAGCAGGATATTC<br>GCTTTGACTCTTTCGGTATTCG	DHA-1 and DHA-2	997	[1]
MultiCaseCIT_for<br>MultiCaseCIT_rev	CGAAGAGGCAATGACCAGAC<br>ACGGACAGGGTTAGGATAGY	LAT-1 to LAT-3, BIL-1, CMY-2 to CMY-7, CMY-12 to CMY-18 and CMY-21 to CMY-23	538	[1]
MultiCaseEBC_for<br>MultiCaseEBC_rev	CGGTAAAGCCGATGTTGCG<br>AGCCTAACCCCTGATACA	ACT-1 and MIR-1	683	[1]
NDM-GBM-F<br>NDM-GBM-R	CCCGGCCACACCAGTGACA<br>GTAGTGCTCAGTGTCGGCAT	NDM	129	[2]
CTX-M-1full_for<br>CTX-M-1full_rev	CTTCCAGAATAAGGAATCCC<br>CGTCTAAGGCGATAAACAAA	CTX-M group 1	949	[3]
CTX-M-9full_for	TGACCGTATTGGGAGTTTG	CTX-M group 9	902	[3]

CTX-M-9full_rev	ACCAGTTACAGCCCTTCG			
aac(6′)-Ib_For	TTGCGATGCTCTATGAGTGGCTA	*aac(6′)-Ib-cr*	482	[4]
aac(6′)-Ib_Rev	CTCGAATGCCTGGCGTGTTT			
mphAF	GTGAGGAGGAGCTTCGCGAG	*mph(A)*	403	[5]
mphAR	TGCCGCAGGACTCGGAGGTC			
QEPfor	TGGTCTACGCCATGGACCTCA	*qepA*	1,137	[6]
QEPrev	TGAATTCGGACACCGTCTCCG			
QnrAm-F	AGAGGATTTCTCACGCCAGG	*qnrA*	516	[7]
qnrA_R	GCCATACCTACGGCGATACC			
qnrB_F	GATCGTGAAAGCCAGAAAGG	*qnrB*	476	[8]
qnrB_R	ATGAGCAACGATGCCTGGTA			
qnrC-F	GGGTTGTACATTTATTGAATC	*qnrC*	447	[9]
qnrC-F	TCCACTTTACGAGGTTCT			
qnrD-F	CGAGATCAATTTACGGGGAATA	*qnrD*	582	[10]
qnrD-F	AACAAGCTGAAGCGCCTG			
QnrSm-F	GCAAGTTCATTGAACAGGGT	*qnrS*	428	[7]
QnrSm-R	TCTAAACCGTCGAGTTCGGCG			
mcr1_320bp_fw	AGTCCGTTTGTTCTTGTGGC	*mcr-1*	320	[11]
mcr1_320bp_rev	AGATCCTTGGTCTCGGCTTG			
mcr2_700bp_fw	CAAGTGTGTTGGTCGCAGTT	*mcr-2*	715	[11]
mcr2_700bp_rev	TCTAGCCCGACAAGCATACC			
mcr3_900bp_fw	AAATAAAAATTGTTCCGCTTATG	*mcr-3*	929	[11]
mcr3_900bp_rev	AATGGAGATCCCCGTTTTT			
mcr4_1100bp_fw	TCACTTTCATCACTGCGTTG	*mcr-4*	1,116	[11]
mcr4_1100bp_rev	TTGGTCCATGACTACCAATG			
MCR5_fw	ATGCGGTTGTCTGCATTTATC	*mcr-5*	1,644	[12]
MCR5_rev	TCATTGTGGTTGTCCTTTTCTG			
mcr-6_mp_fw	AGCTATGTCAATCCCGTGAT	*mcr-6*	252	[13]
mcr-6_mp_rev	ATTGGCTAGGTTGTCAATC			
mcr-7_mp_fw	GCCCTTCTTTTCGTTGTT	*mcr-7*	551	[13]
mcr-7_mp_rev	GGTTGGTCTCTTTCTCGT			
mcr-8_mp_fw	TCAACAATTCTACAAAGCGTG	*mcr-8*	856	[13]
mcr-8_mp_rev	AATGCTGCGCGAATGAAG			
mcr-9_mp_fw	TTCCCTTTGTTCTGGTTG	*mcr-9*	1,011	[13]

mcr-9_mp_rev	GCAGGTAATAAGTCGGTC

References

1. Dallenne, C.; Da Costa, A.; Decre, D.; Favier, C.; Arlet, G. Development of a set of multiplex PCR assays for the detection of genes encoding important β-lactamases in Enterobacteriaceae. *J. Antimicrob. Chemother.* **2010**, *65*, 490-495.
2. Voets, G.M.; Fluit, A.; Scharringa, J.; Stuart, J.C.; Leverstein-van Hall, M.A. A set of multiplex PCRs for genotypic detection of extended-spectrum β-lactamases, carbapenemases, plasmid-mediated AmpC β-lactamases and OXA β-lactamases. *Int. J. Antimicrob. Agents* **2011**, *37*, 356-359.
3. Liu, J.-H.; Wei, S.-Y.; Ma, J.-Y.; Zeng, Z.-L.; Lü, D.-H.; Yang, G.-X.; Chen, Z.-L. Detection and characterisation of CTX-M and CMY-2 β-lactamases among *Escherichia coli* isolates from farm animals in Guangdong Province of China. *Int. J. Antimicrob. Agents* **2007**, *29*, 576-581.
4. Park, C.H.; Robicsek, A.; Jacoby, G.A.; Sahm, D.; Hooper, D.C. Prevalence in the United States of *aac (6')-Ib-cr* encoding a ciprofloxacin-modifying enzyme. *Antimicrob. Agents Chemother.* **2006**, *50*, 3953-3955.
5. Ojo, K.; Ulep, C.; Van Kirk, N.; Luis, H.; Bernardo, M.; Leitao, J.; Roberts, M. The *mef*(A) gene predominates among seven macrolide resistance genes identified in Gram-negative strains representing 13 genera, isolated from healthy Portuguese children. *Antimicrob. Agents Chemother.* **2004**, *48*, 3451-3456.
6. Karczmarczyk, M.; Martins, M.; McCusker, M.; Mattar, S.; Amaral, L.; Leonard, N.; Aarestrup, F.M.; Fanning, S. Characterization of antimicrobial resistance in *Salmonella enterica* food and animal isolates from Colombia: identification of a *qnrB19*-mediated quinolone resistance marker in two novel serovars. *FEMS Microbiol. Lett.* **2010**, *313*, 10-19.
7. Cattoir, V.; Poirel, L.; Rotimi, V.; Soussy, C.-J.; Nordmann, P. Multiplex PCR for detection of plasmid-mediated quinolone resistance qnr genes in ESBL-producing enterobacterial isolates. *J. Antimicrob. Chemother.* **2007**, *60*, 394-397.
8. Kim, H.B.; Park, C.H.; Kim, C.J.; Kim, E.-C.; Jacoby, G.A.; Hooper, D.C. Prevalence of plasmid-mediated quinolone resistance determinants over a 9-year period. *Antimicrob. Agents Chemother.* **2009**, *53*, 639-645.
9. Wang, M.; Guo, Q.; Xu, X.; Wang, X.; Ye, X.; Wu, S.; Hooper, D.C.; Wang, M. New plasmid-mediated quinolone resistance gene, *qnrC*, found in a clinical isolate of *Proteus mirabilis*. *Antimicrob. Agents Chemother.* **2009**, *53*, 1892-1897.
10. Cavaco, L.M.; Hasman, H.; Xia, S.; Aarestrup, F.M. *qnrD*, a novel gene conferring transferable quinolone resistance in *Salmonella enterica* serovar Kentucky and Bovismorbificans strains of human origin. *Antimicrob. Agents Chemother.* **2009**, *53*, 603-608.
11. Rebelo, A.R.; Bortolaia, V.; Kjeldgaard, J.S.; Pedersen, S.K.; Leekitcharoenphon, P.; Hansen, I.M.; Guerra, B.; Malorny, B.; Borowiak, M.; Hammerl, J.A. Multiplex PCR for detection of plasmid-mediated colistin resistance determinants, *mcr-1*, *mcr-2*, *mcr-3*, *mcr-4* and *mcr-5* for surveillance purposes. *Eurosurveillance* **2018**, *23*, 17-00672.
12. Borowiak, M.; Fischer, J.; Hammerl, J.A.; Hendriksen, R.S.; Szabo, I.; Malorny, B. Identification of a novel transposon-associated phosphoethanolamine transferase gene, *mcr-5*, conferring colistin resistance in d-tartrate fermenting *Salmonella enterica* subsp. *enterica* serovar Paratyphi B. *J. Antimicrob. Chemother.* **2017**, *72*, 3317-3324.
13. Borowiak, M.; Baumann, B.; Fischer, J.; Thomas, K.; Deneke, C.; Hammerl, J.A.; Szabo, I.; Malorny, B. Development of a novel *mcr-6* to *mcr-9* multiplex PCR and assessment of *mcr-1* to *mcr-9* occurrence in colistin-resistant *Salmonella enterica* isolates from environment, feed, animals and food (2011-2018) in Germany. *Front. Microbiol.* **2020**, *11*, 80.
